# Olfactory Communication via Microbiota: What Is Known in Birds?

**DOI:** 10.3390/genes9080387

**Published:** 2018-07-31

**Authors:** Öncü Maraci, Kathrin Engel, Barbara A. Caspers

**Affiliations:** Research Group Chemical Signalling, Department of Animal Behaviour, Bielefeld University, Konsequenz 45, 33615 Bielefeld, Germany; k.engel@uni-bielefeld.de (K.E.); barbara.caspers@uni-bielefeld.de (B.A.C.)

**Keywords:** birds, olfaction, odour, social communication, microbiota, skin, uropygial gland, feathers, gut, chemical signalling

## Abstract

Animal bodies harbour a complex and diverse community of microorganisms and accumulating evidence has revealed that microbes can influence the hosts’ behaviour, for example by altering body odours. Microbial communities produce odorant molecules as metabolic by-products and thereby modulate the biochemical signalling profiles of their animal hosts. As the diversity and the relative abundance of microbial species are influenced by several factors including host-specific factors, environmental factors and social interactions, there are substantial individual variations in the composition of microbial communities. In turn, the variations in microbial communities would consequently affect social and communicative behaviour by influencing recognition cues of the hosts. Therefore, microbiota studies have a great potential to expand our understanding of recognition of conspecifics, group members and kin. In this review, we aim to summarize existing knowledge of the factors influencing the microbial communities and the effect of microbiota on olfactory cue production and social and communicative behaviour. We concentrate on avian taxa, yet we also include recent research performed on non-avian species when necessary.

## 1. Introduction

Are body odours just the by-products of our physiology and poor personal hygiene or are they valuable signals produced by bacteria, providing cues to other individuals about ourselves? The sweaty scent of human armpits is actually not the smell of the sudoriferous secretion, but the result of commensals on our skin, digesting its compounds into volatile, odorous molecules [1]. Whether or not this scent can be called an olfactory signal serving in communication, remains however speculative. Nevertheless, the finding that microbes are present in specific scent glands in mammals and are involved in modulating specific odours led to the hypothesis that microbes play an important role in communication [2,3,4]. Indeed, animals harbour complex and diverse microbial communities on different body parts such as skin, hair or feathers, in the gastrointestinal, respiratory and reproductive tracts. These communities are dominated by bacteria, but also consist of fungi, archaea, protozoa and viruses [5]. Although some of these microbes are associated with parasitism, many microbes are beneficial for their hosts, fulfilling crucial physiological functions [6,7].

Apart from its physiological functions, accumulating evidence has revealed that microbiota can modulate the hosts’ behaviour across many animal taxa (reviewed in [2,3,4,8]), for example, through the development or modulation of personality [9], by being involved in individual recognition [10,11] and/or mate choice [12,13,14]. Although the exact details of the interplay between microbiota and host behaviour has not been fully discovered yet, one of the plausible mechanisms is that olfactory signals produced by microorganisms might influence the hosts’ behaviour [15,16,17]. This idea was postulated in the late 70s as the “fermentation hypothesis of chemical recognition”, which states that microbially-derived olfactory molecules mediate social communication in mammals [11,18]. Based on this hypothesis, microbial communities produce odorant molecules as metabolic by-products and thereby modulate the biochemical signalling profiles of their animal hosts. As the composition of the symbiotic microbiota is influenced by several factors (including the phylogeny of the host, life-history traits such as age and sex, the genetic structure of the host, social interactions, and environmental variables such as diet and habitat), microbe-specific odorants might transfer this information about the host. This information might thus be very difficult to fake and is likely to be an honest signal in intraspecific communication.

Recent developments in molecular survey techniques have enabled scientists to investigate host-microbiota interactions from different aspects. Nevertheless, several questions about the mechanisms underlying microbial-based bio-chemical signalling and interactions between microbiota and host behaviour remain elusive in this nascent field. Here, we aim to summarize existing knowledge of the impact of microbiota on olfactory communication systems.

In the first part of this review, we summarize evidence of microbe induced odours and then we address factors influencing the structure of the microbial communities. Further, we discuss potential functions of microbe-induced odours and the effect of symbiotic communities on mate choice, social and communicative behaviour. We focus on avian taxa, yet the vast majority of microbiome studies have been conducted in non-avian species such as mammals and insects, leaving bird microbiota relatively under-investigated. Considering these studies can provide a basis for further investigations of avian microbiomes, we also include recent research performed on non-avian species when necessary. We will end our review discussing potential evolutionary implications of microbe induced odours and the advantage of studying birds.

## 2. Potential Body Regions of Microbe Induced Odour Production

Most of our knowledge of microbe-induced odours comes from mammals [19]. The fact that germ free reared rats, for example, do not show any individual-specific odour anymore [20] led to the conclusion that microbes are involved in social communication by producing a host-specific body odour. Well accepted is the production of olfactory cues by microbiota in specialized skin pouches, for example around the anus of carnivores. This link has been discussed and demonstrated in red foxes and lions [21], mongooses [10], meerkats [17,22], European badgers [23] and hyenas [16,24]. However it is important to mention that all of these studies are correlational. For example, in hyenas, both, the chemical composition of the anal gland, as well as the composition of microbes within this gland, are affected by sex and reproductive status in both species investigated, implicating that odour cues produced by microbiota in the scent pouches communicate information related to their hosts [16]. Sac-winged bats (*Saccopteryx bilineata*) harbour bacterial communities in their wing sacs (skin pouches with odouriferous content on males’ wings that are used in courtship), which they clean and refill with secretions from various body regions every day [25]. Voigt and colleagues [25] suggest that male sac-winged bats thereby control the bacterial growth and the grade of fermentation of the secretion in their skin pouches, which enables them to maintain an individual odour. An impact of bacteria in intraspecific social communication has been shown in wild (*Cavia aperea*) and domestic guinea pigs (*Cavia porcellus*), in which the perineal glands harbour large numbers of bacteria and males preferred secretions containing bacteria over sterile secretions [26].

While there is quite a substantial body of evidence that microbes influence odours and social interactions in mammals, studies in birds are still scarce, although there is no reason to assume that microbes might not be involved in odour production in birds.

One body site that is probably involved in body odour production is the uropygial gland [27,28], dorsally located and a bird’s main secretory skin gland. While preening, birds distribute the secretion of this gland all over their feathers with their bill [29]. The secretions have been shown to contain bacteria, at least in some species (hoopoes (*Upupa epops*) [30], dark eyed juncos [31]) and an example from red-billed woodhoopoes (*Phoeniculus purpureus*) showed that the secretion of the preen gland has been less odourous after the injection of an antibiotic [32]. Similarly, Martín-Vivaldi and colleagues [30] showed that the chemical composition of the European hoopoe preen secretion is under the influence of symbiotic bacteria and the abolishment of these bacteria by an antibiotic treatment alters the composition of volatiles drastically. However, the existence of bacteria in hoopoe preen gland secretions is usually discussed in its probable role as a defence mechanism against predators and feather degrading bacteria or maladaptive eggshell microbes, rather than intraspecific communication. Whittaker and Theis [31] identified in dark-eyed junco (*Junco hyemalis*) uropygial gland secretions bacteria that belong to genera which comprise known odour producers. On the other hand, it must be acknowledged that Whittaker and colleagues [33] could not confirm a significant covariation between bacterial communities and the volatile profiles of preen secretions.

Studies on bird feathers show that they harbour a substantial amount of bacteria on their surface [34,35] with some of them being capable to degrade the feather substance [36]. Microbiota may be regulated by substances deriving from the uropygial gland secretion ([37], reviewed in [38]) and feather-degrading bacteria could be involved in odour production via breaking down feather components into small, possibly volatile, fragments. Because of their extended surface, feathers offer a great surface for odours to emerge from a bird, however, to the best of our knowledge, thus far no study has investigated this possibility.

Another potential pathway of odour production that has largely been ignored is the involvement of skin bacteria in odour production. As mentioned above, studies in humans have shown that the malodour of sweat comes from specific bacteria in the armpit [1]. Furthermore, olfactory cues from human feet are known to attract *Anopheles* flies. Individuals that showed skin emanations attractive to the flies showed significantly higher bacterial loads on their feet and could be distinguished in the abundance of certain bacterial taxa, indicating the production of olfactory cues by skin microbes [39]. Both studies indicate that skin microbiota play a role in odour production. Studies in birds are still rare, but Engel and colleagues [40] showed in three different passerine bird species that bacterial communities on two different locations (neck and skin around the uropygial gland) of each individual were more similar to each other than to the samples of other individuals of the same species. This suggests, at least in some passerine bird species, an individual microbial profile on the skin. Although the avian skin does not possess sebaceous glands except for the prominent uropygial gland, it has been shown to harbour sebokeratinocytes that release lipids to the bird’s skin surface [41]. These lipids may control the growth of resident bacteria or serve as nutrients for skin surface microbiota [42] that produce body odours. Olfactory cues are known to be used in zebra finches to avoid related individuals when choosing a mating partner [43] and, consequently, if we assume bacteria to play a role in recognition, then individuals might be distinguished and chosen by their unique bacteria-derived odour profile. A similar result has been found in two species of larks, in which the bacterial community of the brood patch, feathers and the cloaca could be explained to a large amount of by the individual ID [44]. In contrast, female leach´s storm petrels seem to differ between body sites, i.e. the brood patch and the uropygial gland [45].

Whereas some of the above-mentioned studies suggest an impact of body surface microbes on odour production, the impact of gut microbes on odour production has hardly been explored in vertebrates. Most of our knowledge about the interactions between gut microbiota and scent production comes from insect studies (reviewed in [4]). For example, in desert locusts (*Schistocerca gregaria*) odour profiles of faecal pellets are affected by a pheromone (Guaiacol) which is produced by indigenous gut bacteria [46,47,48]. Similarly, a contribution of gut bacteria in olfactory-based chemical communication was shown in another insect species, the German cockroach (*Blattella germanica*) [49]. Similarly, in mice the synthesis of trimethylamine, an odorous volatile compound found in mice urine influencing attractiveness to conspecifics, is associated by gut microbial metabolism [19].

Investigations of the microbiota in the digestive tract of birds focus, to our knowledge, on commercial poultry. Studies have shown that adequate nutrition can reduce the odour emissions of broiler faeces i.e. the components occurring in faeces acting as substrate for certain bacteria [50]. This influence of food intake on odour could lead to the conclusion that faeces of birds communicate the health status of an individual, meaning that less odourous droppings belong to animals with a balanced enteric flora. In another study concerning gut microbiota, the odours of certain strains of Enterobacteriaceae, derived from faeces of chicken and barn swallows, have been shown to attract apple maggot flies, *Rhagoletis pomonella* [51]. As body odours are partially consisting of by-products of every day metabolic processes, one can assume that gut microbes might also be involved in shaping individual-specific body odours. Whether avian faecal odours are important in social communication remains to be investigated. For example, faecal odours could act as territorial signals around nests and roosting sites. However, we assume that if at all, these are probably more important in ground living bird species, in which the faeces can be directly linked to an individual bird. A highly interesting connection between faeces and predator deterrence has been demonstrated in eider ducks. Females defecate over their eggs when disturbed from the nest and the faeces seemed to deter crows from predating on those eggs, probably through their strong smell. As some birds defecate around their nest (e.g., the colony-breeding sandwich tern, *Sterna sandvicensis*), it seems possible that this behaviour acts as some kind of territory marking, potentially deterring intruding neighbours or enabling nest recognition. Sandwich terns lay rather light-coloured eggs and the faeces have been considered to provide a similar-coloured background for these eggs [52] but it is imaginable that this accumulation of feaces also serves for some kind of olfactory communication. Such a mechanism might be responsible for nest recognition in zebra finches, in which fledglings are able to recognise their nest by smell [53]. At the moment this is pure speculation, but similar to what is known in mammals [54,55] faecal marking might also be used in birds, e.g., in lekking species, for chemical communication.

It must be considered that most studies linking microbiota and odour are correlational and empirical data is often absent. Apparently, more studies investigating the link between odour-producing bacteria, body odours and behaviour would help understanding the role of gut and surface bacteria in bird communication. In the next sections we will review the existing literature about the host specific factors that are involved in determining a host-specific microbe community. These factors bring us to the conclusion that microbe-influenced olfactory cues are qualified to transfer fitness relevant information.

## 3. Sources of Microbial Diversity in Birds

There are various factors influencing microbial diversity in animals, resulting in a complex interplay between host-specific factors, environmental conditions and social interactions. For example during early development, animals acquire bacteria via vertical transfer from their mothers, or horizontal transfer from the surrounding physical and social environment. In this section we will investigate known factors contributing to the microbial composition in birds (for a summary see Table 1).

### 3.1. Host-Specific Factors

Phylogeny of the host significantly affects the composition of microbiota [5]. From an evolutionary point of view, this is not surprising as similar organisms have similar life-history traits. Furthermore there is congruence between the evolutionary histories of host and microbial communities. This can be explained by phylosymbiosis, which refers to “an eco-evolutionary pattern in which evolutionary changes in the host associate with ecological changes in the microbiota” [56,57]. Based on phylosymbiosis, the evolutionary history of microbiomes across host species maintains an ancestral signal of the host’s evolution [58]. Prominent differences in cloacal microbial assemblages were documented in great spotted cuckoos (*Clamator glandarius*), a brood parasite and its host species, magpies (*Pica pica*) [59]. By analysing the gut microbiota of 59 Neotropical bird species [60], showed that the taxonomic identity of the host had a greater impact on microbial diversity compared to other ecological variables. A comprehensive study investigating the gut microbial composition in 51 passerine species documented significant differences at the interspecific level [61]. Waite and Taylor [62] found in a meta-analysis that the host taxonomy is the most important determinant of the gut microbial diversity. Investigation on gut microbial communities in eight wild Neotropical bird species uncovered that bird taxonomy is an important factor predicting community structure [63]. Similarly, investigations of gut microbiota in four penguin species revealed that both, the composition and relative abundance of microbial species are influenced by the taxonomy of the host [64]. Moreover, a species-specific gut microbiota was documented in red knot (*Calidris canutus*) and ruddy turnstone (*Arenaria interpres*) occupying environmentally similar habitats [65]. In line with this, a recent study showed that three different estrildid finch species showed species-specific microbe communities on their skin [40], bearing the potential that microbes might be involved in species-specific body odours [66]. In line with this, a comparative study among songbird species revealed that feather waxes encode information about the species and the phylogeny [67]. Here, a comparative study correlating odours and microbe communities among various bird species could gain insight that is currently missing.

Age or the developmental status of the host is another determinant: During the early stages of life, bacterial communities are unstable, and the diversity is quite low compared to adulthood [68,69]. This can be attributed to the influence of reproductive hormones potentially relevant for shaping the bacterial communities [17]. Another explanation might be the transmission from parent-dependent feeding strategies to independent feeding and accompanying dietary changes, which create new niches leading to bacterial succession towards more diverse and complex bacterial ecosystem [70]. For example the bacterial community structure of the gut becomes more complex as the chickens matured [71,72]. Similarly, in the hoatzin (*Opisthocomus hoazin*) [70] and black-legged kittiwakes (*Rissa tridactyla*) [73] the profile of gut microbiota changes across different development stages.

The microbial diversity in animals is also determined by the host’s sex, as it strongly affects physiology and behaviour [74,75,76,77,78]. Hormonal differences and accompanying alterations in the immune system between males and females are probably the underlying mechanism for sex-specific differences in microbiota [75]. An impact of sex on the composition of microbe communities has been demonstrated in brown-headed cowbirds (*Molothrus ater*) [79], broiler chickens [80,81], and in leach’s storm petrels [45], whereas an impact of sex was not observed in barn swallows, *Hirundo rustica* [82] estrildid finch species [40] and dark-eyed juncos [31,33]. Interestingly, in hoopoes, the prevalence of Enterococcus bacteria in the uropygial gland of breeding females and nestlings is significantly higher than the one of males and non-breeding females, indicating reproductive status might be another important factor in terms of microbial diversity [83].

Another possible factor contributing to the composition of microbes in animals is host genetics [84,85,86] (see [3] for a review on gut microbes). For example in humans, gut microbe community similarity correlates with genetic relatedness [86]. Interestingly, studies conducted on different bird species revealed contradicting results. For example, in adelie penguins (*Pygoscelis adelie*), genetically related individuals have very similar gut microbe communities even if they inhabit distant geographical areas [87]. Similarly, the hosts’ genetics and sex are the major determinants of gut microbiota in chickens [88]. Nevertheless, a substantial number of studies suggests that the impact of genetic factors to patterns of microbial composition is smaller than those of external factors (reviewed in [89,90]). For example, in barn swallows [79], zebra finches (*Taeniopygia guttata*) [91] and brown-headed cowbirds [82] the structuring of the cloacal microbiota is related to environmental factors and social interactions and outweighs the impact of the hosts genetics. Similarly, investigating both cloacal and uropygial microbiota in dark-eyed juncos Whittaker et al. [33] documented that social environment has more prominent impact on microbial diversity than genetic relatedness.

Although the genes and the exact mechanisms responsible for the determination of host-specific microbiota have not been identified yet, one promising candidate region is the major histocompatibility complex (MHC). The MHC is an essential part of the adaptive immune system that codes a set of cell surface proteins involved in discriminating self from non-self and is therefore a predestined candidate to be involved in shaping the microbe community of the host. The MHC molecules specifically bind to foreign microorganisms to present them for immune recognition, thereby mediating the hosts’ immune response to pathogens. Indeed, MHC variation correlates with the non-pathogenic gut microbial composition in mice [92], rats [93], fish [94] and humans [95]. In birds, the relationship between the MHC genotype and composition of microbiota is less well investigated. A recent study revealed that in leach’s storm petrels bacterial community structure in the uropygial gland varied between homozygous and heterozygous males for *DAB2* gene of MHC class II antigen [45]. However, the authors noted that the sample size of the study is too small to cover the population-level diversity of MHC genotypes in the study species. Considering its function in the determination of microbiota, the MHC seems to be an important genetic component determining microbial diversity. However, to our knowledge, there is no study convincingly proving the relationship between MHC genotype and microbial diversity in avian taxa.

### 3.2. Environmental Factors Influencing Microbiota

Environmental factors are one of the main drivers in shaping the gut microbiota, sometimes overweighing genetic factors [87,96,97,98,99]. Host geography and accompanying alterations in habitat such as climate, flora and fauna influence the pool of microorganisms in the local environment [61] and consequently, have prominent effects on microbial composition of the host. For example, Banks et al. [87] show that adelie penguins living in close geographical proximity have similar gut microbe communities. Microbial assemblages present in the cloacae of spotted towhee (*Pipilo maculatus*) correlated with the geographic origin of the birds [100]. In brown-headed cowbirds (*M. ater*), a generalist brood parasite, sampling locality is the most important factor explaining gut microbial structuring [79]. Considering horizontal transfer from the surrounding environment during the early stages of live is one way how animals acquire microbial communities [101], the nestling environment is an important factor characterising microbial diversity in birds. For example, employing a cross-fostering experiment between great tits (*Parus major*) and blue tits (*Parus caeruleus*) [102] it was shown that sharing the nest has more prominent effects in determining microbial diversity compared to host’s taxonomic identity. Similarly, in sympatric living woodlarks (*Lullula arborea*) and skylarks (*Alauda arvensis*), nest identity is the most important factor in terms of explaining microbial diversity in cloaca, brood patch skin and feathers [44]. In line with this in the dark-eyed junco, nest identity is a good predictor of both cloacal and uropygial microbiota [33].

Furthermore, hoopoes seem to be able to acquire uropygial gland symbionts from the environment during the development of their gland. This has been shown by a cross-foster experiment among hoopoe nests and a second experiment between hoopoes and an unrelated bird species, the great tit [103]. Intriguingly, hoopoe nestlings seemed to obtain their uropygial gland bacterial communities rather from the host nest than from the nest of origin (thus their parents) and also independent of contact to their own species. The latter could hint for a host-specific factor, meaning that the hoopoe uropygial gland in particular offers an environment for the symbionts (here: *Entereococcus* spp.). The composition of skin microbes seems to be largely environment-driven [104] with nests being a substantive source of microbiota on nestlings’ skin [105]. Thus, odours may carry important information about the environment an individual is living in and thus may be important in social communication.

The composition of gut microbiota is also highly influenced by the host’s diet [5,85]. There is an extensive variation among avian taxa in terms of food source, spanning from seeds to carrion and the effect of diet on patterns of gut microbiota was shown in different avian taxa [60,106,107,108]. Turkey vultures (*Cathartes aura*) are one of the most striking examples of how the food influences host’s gut microbiota [109]. The hindgut of these scavengers predominantly contains Clostridia and Fusobacteria, which are pathogenic to other vertebrates. These toxic bacteria are not only tolerated by vultures, but also allow them to digest carrion. Furthermore, new world vultures harbour a very special bacterial community on their facial skin. The microbiota probably originates from the carrion the vultures consume and therefore can be seen as acquired from the diet and the environment [109]. Very probably, these microbiota somehow contribute to the body odour of vultures, but whether this serves for communication or if it is just an accompaniment of the diet remains unclear.

### 3.3. Social Interactions Influencing Microbiota

Social and sexual contacts mediate acquisition and exchange of microbes between group members and mates, shaping the composition and structure of microbial communities in social animals [98,110,111,112,113,114]. Although this mechanism might mediate spreading pathogenic microorganisms, it also ensures the transfer of symbionts between group members providing a benefit to social animals [3,115]. In many bird species, the hatchlings are fed on regurgitated food by their parents, allowing vertical transmission of microbes. Furthermore, consumption of adult faeces by juveniles was reported in ostriches which might facilitate microbial colonization of the gut [116]. Microbes can also be transmitted between group members via allopreening, particular present in social bird species. Thus we can speculate about the possibility that group membership might be encoded via socially transmitted microbes. In line with this, in dark-eyed juncos the paired males and females have a similar preen gland bacterial community composition [31].

Furthermore, birds have a special organ, the cloaca, which has functions in both excretion of faeces and urine and transfer of gametes, allowing the transfer of the microbial organisms between couples, during copulation [117]. In order to estimate the extent of social transmission in birds, Kulkarni and Heeb [91] experimentally inoculated a specific strain of *Bacillus licheniformis* on the feathers and cloaca of zebra finches. They observed that bacteria were transferred between the birds, probably via oral-faecal-genital route: *B. licheniformis* transmission most likely occurred from beak to gut during preening and from cloaca to cloaca during mating. It is worth noting that transmission from males to females was more frequent. The structure of cloacal microbiota showed substantial correlations between mated couples of house sparrows [118]. A transfer of microbes between mated couples was also noted in red-winged blackbirds (*Agelaius phoeniceus*) [119] and black-legged kittiwakes [117]. Nevertheless, in black-legged kittiwakes the female cloaca has a resilient mechanism that destroys mate-shared bacteria and reverts the microbial profiles to pre-mating state [117].

To summarise, similar to other taxa, the establishment and maintenance of microbiota in birds are driven by complex interplays between host-specific factors, environmental conditions and social relationships (Table 1). Based on studies summarised in previous parts, the surrounding environment, the diet and the social interactions are the major determinants of an individual’s microbial composition. Influence of host-specific factors such as taxonomy, age, developmental state, sex and genetics is also evident in different avian taxa. However, the question of what is the relative contribution of host-genetics compared to environmental factors still lacks an answer. Disentangling heritable factors from environmental ones is specifically important considering the potential involvement of microbes to chemical communication: In order to provide an honest signal about host-traits at least part of the microbiota need to be determined by the genotype. Therefore, it is important to understand the extent to which hosts can regulate the establishment and maintenance of microbial composition and potential mechanisms allowing such a control. Due to the difficulty to disentangle the heritable factors from external factors in many species, here, birds provide an advantage by enabling scientists to separate maternal from environmental factors through manipulating the prenatal environment. Moreover, it is still unclear to what extent smaller differences in microbe communities are detected by other individuals during social interactions. Here more studies are needed to answer questions about the importance of environmental factors, social interactions and the hosts’ genes. In particular we need experiments modulating one of these factors while keeping constant the other factors.

## 4. Functional Aspects of Microbe Induced Odours in Social Communication

Considering the composition and structure of the microbial communities are influenced by host-traits such as phylogeny, social interactions, diet and health status, microbes may broadcast information about the host individuals and therefore be used in social communication [8]. This relationship is quite obvious and well documented for certain infections, which come along with specific odours in humans and other mammals [120,121]. This information about the current health status is very important and can alter mate choice decisions in mice [122]. Olfactory signals made by gut microbiota also influence mate-choice decisions in different insects (reviewed in [123,124]). One of the most striking studies relating gut microbiota to mate choice was conducted in *Drosophila melanogaster* [14], in which diet-specific differences in gut microbe compositions induced differences in cuticular hydrocarbon (CHC) composition. In another study, the effect of genetic relatedness, familiarity and diet on mating decisions of three *Drosophila* species was addressed [125]. All three species preferred to mate with familiar partners and two of the species invested more in partners reared on the same diet. Interestingly, the removal of gut bacteria by antibiotics abolished the influence of familiarity and diet type on mate choice. Similarly, German cockroaches that lacked bacteria in the alimentary tract were shown to have lower concentrations of volatile carboxylic acids in their faeces, resulting in decreased attractiveness to conspecifics [49]. Inoculation of bacteria normalised this defect and the degree of attractiveness was correlated with bacterial diversity. All these examples show that microbe-induced odours are involved in social communication. However whether this is also the case in birds is still unknown and needs further investigations.

Apart from their functions in mate attraction and mate choice, gut bacteria seem to function in recognition of conspecifics and nestmates in termites [126,127] and kin in *D. melanogaster* [128] via production of chemical cues. In red harvester ants (*Pogonomyrmex barbatus*), an experiment conducted by Dosmann and colleagues [129] showed that ants externally treated with a bacterial suspension were rejected by their nestmates as if they were not members of the colony. Ants treated with antibiotics, on the other hand, were not rejected. Dosmann and colleagues [129] assumed that the addition of a foreign bacteria-produced odour plays a stronger role in recognition than the lack of a familiar odour produced by bacteria (the CHC profiles were assumed to not being altered by the treatment). Consequently, cuticular microbiota really seem to be involved in odour production and nestmate recognition in red harvester ants.

Microbes are also supposed to be involved in recognising groups of specific individuals in mammals. This can be explained by group members harbouring more similar bacterial communities due to transfer of microbes and being exposed to similar ecological conditions [24]. This may enable individuals to recognise group members by microbially produced volatiles. The evidence of the contribution of gut microbiota in recognition of conspecifics comes from a mouse study: bacterially-produced scent molecules provide information about the taxonomic identity of the host, attracting conspecifics and repelling other rodents [19]. In hyenas, anal pouch bacteria are highly structured among clans, mediating odour-based recognition of group members [24,130]. In order to allow group identification based on microbe induced odours, new group members need to actively overmark fresh scent marks or need to be actively involved in group rituals, in order to transfer group-specific microbe communities [24,130].

**Figure 1:**
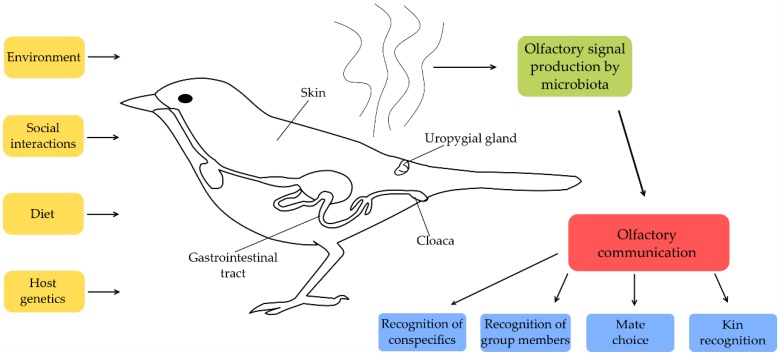
Possible factors involved in shaping microbiota-based communication.

To our knowledge, there are no studies investigating experimentally whether the found association between microbe and olfactory signals [31,33] is used in social communication of birds. However, considering birds use olfactory cues for a variety of purposes [131,132,133,134], it is feasible that olfactory signals produced by microbiota are used in different aspects of social communication (Figure 1). Therefore, further studies should attempt to address the interaction between gut, uropygial gland and surface microbiota, olfactory signalling and communicative behaviour in avian taxa.

## 5. Evolutionary Implications and Future Prospects

As shown above, there is a substantial body of evidence showing that animal-associated microbes produce odorant substances, determined by the diversity and composition of microbial species. Bacterial communities are shaped by several factors (see above) and therefore, microbe-induced odours might transfer fitness relevant information important for other individuals that is difficult to manipulate by the host. Accumulating evidence already implies that these olfactory cues are involved in several microevolutionary processes including different aspects of social communication, such as recognition of conspecifics, group members, individuals and kin. Due to a lack of knowledge we can only speculate about the evolutionary implications, but if bacterial communities are under selection and the odours being produced are important in social communication, then we should consider calling microbe-induced odours signals instead of cues, evolved also for the purpose of information transfer.

Nevertheless, a lot of questions about the interplay between microbes, olfactory signal production and host communicative behaviour are unexplored yet. For example, casual connections between individual differences in microbiota and communicative behaviour of their hosts have not been discovered yet. Another important question is how common and reliable the microbiota-mediated olfactory odorants are across different animal taxa. In order to understand the significance of microbially induced odour cues in the evolution of host species, it is necessary to establish the mechanisms enabling the transfer of symbiotic microorganisms across generations which raise several questions about heritability of microbiota. As explained in the previous sections, animals acquire their microbes via two main mechanisms: First, from parents and other community members. This seems a reliable mechanism to ensure trans-generational transfer of microbiota and co-diversification of animal hosts and their microbes. The second mechanism of acquisition of microbes is horizontal transfer from the surrounding environment. Therefore, from an evolutionary point of view, it is important to disentangle environmental factors from host-specific factors, most importantly host genetics, in order to understand whether individual microbial communities are a trait of an animal itself, rather than an artefact of the environment. This information would allow us to evaluate the honesty of signals produced by microbial communities. Here, birds are promising organisms for several reasons.

Firstly, birds have a sense of smell and use olfaction for a variety of reasons [131,132,133].

Secondly, birds use secretion from the uropygial gland to preen their feathers, which is most likely involved in both, olfactory communication [135,136,137] and maintaining the stability of the surface microbial community [37,138]. This gives the possibility for microbial interactions at different stages and locations and for selection to act at different stages i.e., already in the preen gland, on the feathers, skin, gut and/or cloaca. These are all locations harboured by microbes and underlying different selective forces.

Thirdly, birds are oviparous (egg-laying) organisms, which make them ideal study subjects for microbiota-mediated olfactory signalling studies compared to viviparous (birth-giving) organisms, in which the close mother-offspring relationship makes it difficult to disentangle environmental from genetic factors in shaping the composition and structure of microbiota. This is in contrast to birds, which lack a direct association between mother and the offspring during embryonal development, which prevents in vivo vertical transmission of microorganisms. Although it was shown that bird egg surfaces contain microorganisms at or shortly after egg laying [138,139] and the composition of these microbes are similar to those of female’s cloaca [140], uropygial gland [141], skin and body feathers [44] as well as the nest material [104,142,143], it is still elusive whether eggs are a crucial step in the transfer of maternal or environmental microbes to the embryo before hatching. Therefore, oviparous animals are ideal to disentangle the impact of environment and genetics by allowing to manipulate the prenatal environment.

## 6. Conclusions

In conclusion, birds are ideal study objects to investigate gene–environment interactions involved in microbe specific communication systems. The growing evidence that birds indeed make use of olfactory social communication together with the ability to manipulate the hatching environment and separating this from related individuals makes birds a promising study species to investigate mechanisms and functions of microbe induced olfactory communication.

## Figures and Tables

**Table 1:** Factors influencing microbiota in birds. Only studies confirming a positive link are included.

	Study Species	References
**Host Environment**		
**Nest Environment**	Great tit (*Parus major*), blue tit (*Parus caeruleus*)	[102]
	Woodlark (*Lullula arborea*), skylark (*Alauda arvensis*)	[44]
	Dark-eyed junco (*Junco hyemalis*)	[33]
	Hoopoe (*Upupa epops*)	[103]
	Pied flycatcher (*Ficedula hypoleuca*)	[105]
**Habitat/Geographic Proximity**	Adelie penguin (*Pygoscelis* *adeliae*)	[87]
	Towhee (*Pipilo maculatus*)	[100]
	Brown-headed Cowbird (*Molothrus ater*)	[79]
	Comp. analysis on 59 neotropical bird species	[60]
	Zebra finch (*Taeniopygia* *guttata*)	[91]
**Social Environment**	Dark-eyed junco (*J. hyemalis*)	[31]
	Zebra finch (*T. guttata*)	[91]
	Black-legged kittiwake (*Rissa tridactyla*)	[117]
	Red-winged blackbird (*Agelaius phoeniceus*)	[119]
	House sparrow (*Passer domesticus*)	[118]
	Barn swallow (*Hirundo rustica*)	[82]
**Host-Specific Factors**		
**Host Taxonomy**	King penguin (*Aptenodytes* *patagonicus*), gentoo penguin (*Pygoscelis* *papua*)*,* macaroni penguin (*Eudyptes* *chrysolophus*), little penguin (*Eudyptula* *minor*)	[64]
	Red knot (*Calidris canutus*), ruddy turnstone (*Arenaria interpres*)	[65]
	Zebra finch (*T. guttata*), diamond firetail (*Stagonopleura* *guttata*), Bengalese finch (*Lonchura striata domestica*)	[40]
	Magpie (*Pica pica*), great spotted cuckoo (*Clamator glandarius*)	[59]
	Comp. analysis on 59 neotropical bird species	[60]
	Comparative study on 51 bird species	[61]
	Metaanalyses	[62]
	Comparative analyses on 8 neotropical bird species	[63]
**Host Genetics and Genetic Similarity**	Chicken (*Gallus gallus*)	[88]
	Adelie penguin (*P. adeliae*)	[87]
**Age/Reproductive Status**	Hoatzin (*Opisthocomus hoazin*)	[70]
	Chicken (*G. gallus*)	[71,72]
	Kittiwake (*R. tridactyla*)	[73]
	Hoopoe (*U. epops*)	[83]
**Sex**	Brown-headed cowbird (*M. ater*)	[79]
	Chicken (*G. gallus*)	[80,81]
	Leach’s storm petrel (*Oceanodroma leucorhoa*)	[45]
